# Burden and Correlates of Geriatric Depression in the Uyghur Elderly Population, Observation from Xinjiang, China

**DOI:** 10.1371/journal.pone.0114139

**Published:** 2014-12-01

**Authors:** Lei Feng, Ping Li, Chen Lu, Weiming Tang, Tanmay Mahapatra, Yu Wang, Xihua Wang, Ying Ma, Yanli Ben, Xiaolin Cao, Sanchita Mahapatra, Min Ling, Anshuan Gou, Yanmei Wang, Jiangqin Xiao, Ming Hou, Xiuli Wang, Bo Lin, Ruoling Chen, Faxing Wang, Zhi Hu

**Affiliations:** 1 The people's hospital of Xinjiang Uyghur autonomous region, Urumqi, Xinjiang, 830000, China; 2 University of North Carolina Project-China, Guangzhou, Guangdong, 510095, China; 3 Fielding School of Public Health, University of California Los Angeles, Los Angeles, CA, 90066, United States of America; 4 Anhui medical university, Hefei, Anhui, 230032, China; 5 Centre for Health and Social Care Improvement (CHSCI), Faculty of Education, Health and Wellbeing, University of Wolverhampton, Wolverhampton, WV1 1DT, United Kingdom; University Heart Center, Germany

## Abstract

**Background:**

With the gradual aging of the population, geriatric depression has become a major public health issue in China owing to its overall upward trend and associated negative socio-economic impact. Dearth of information regarding the burden and correlates of geriatric depression among Uyghur minority population in Xinjiang Autonomous Region, called for a comprehensive survey involving representative sample for designing efficient targeted intervention to control this disabling disease.

**Methods:**

A cross-sectional study was conducted among 1329 consenting Uyghur elderly in 2011 in six randomly selected communities/villages in Xinjiang. Information about socio-demographics, behavior, negative life-events, satisfaction regarding income/quality of life and other chronic diseases were collected while assessment of geriatric depression was done using Geriatric Mental State Schedule (GMS).

**Results:**

Among these participants, majority were currently married, had attended elementary school or less, had an average annual family income of less than 3000 Yuan/person, had strong religious beliefs while 10.61% (2.77% in urban and 23.60% in rural area) had geriatric depression (5.91% among male and 14.58% among females). 61.83% were suffering from other chronic diseases, 96.16% could take care of themselves and 39.28% had experienced negative events during last two years. Religious belief (AOR = 3.92, 95% CI 1.18–13.03), satisfaction regarding quality of life (AOR = 0.53, 95% CI 0.37–0.84) and income (AOR = 0.75, 95% CI 0.35–1.60), suffering from more chronic diseases (AOR = 1.70, 95% CI 1.42–2.04), experiencing three or more negative events (AOR = 1.72, 95% CI 0.92–3.22) and lack of ability to take self-care (AOR = 2.20, 95% CI 1.09–4.48) were all associated with having geriatric depression with or without adjustment for gender, education and occupation.

**Conclusion:**

High prevalence of geriatric depression among Uyghur elderly in Xinjiang seemed to call for urgent interventions, specifically targeting rural residents, who experienced more negative life-events, were suffering from chronic diseases and were dissatisfied with their income and quality of life.

## Introduction

It was estimated that about 22.7% of the citizens of China will be aged 65 or more within 2050 [1]. With this gradual aging of the population, increasing burden of non-communicable diseases has become a major public health concern in this country [2]. Among these chronic diseases, mental health issues were often overlooked, particularly among elderly [3], in spite of being one of the main reasons for suffering and disability in this population [4].

The cardinal manifestations of mental health problems among aged persons include geriatric depression, anxiety and psychomotor retardation [5]. Among these psychiatric disorders, geriatric depression seemed to be posing a worrisome public health threat as evidenced from the upward trend of its incidence and prevalence with advancing age [6], [7] and overall [8]. Increasing burden of other chronic diseases, cognitive impairment and resultant depression might have contributed in further worsening of the scenario among elderly [9]. Owing to the associated disability, suffering and suicidal tendencies, this increasing occurrence of geriatric depression together with the phenomenon of rapid aging in Chinese population may well culminate into a huge socio-economic burden on the Government, society, families and individuals in near future if not controlled urgently.

Contemporary available literature also highlighted huge disparities between the residents of urban and rural areas of China in terms of socio-demographics, economic factors, health awareness, healthcare-seeking along with social and infrastructural support. [10], [11]. In 2012, the average family income in China's urban areas was about $2,600 (≈16412 Yuan), while it was $1,600 (≈10100 Yuan) in rural areas [12]. These disparities were likely to be more pronounced in the relatively underdeveloped areas like: Xinjiang Autonomous Region and among the ethnic minority groups like Uyghurs.

Xinjiang Autonomous Region is the largest administrative division of China and 46% of the total population here belonged to Uyghur ethnic minority group [13]. In 2010, the proportion of persons aged 60 years or more was estimated to be 10.93% in Xinjiang [13]. In this region, the rural population was more aged than urban while Uyghur minority groups were older than residents of Han ethnicity [14].

Very few research had ever been conducted on mental disorders among Uyghur population [15]. Among those, observations pertaining to the elderly were even less while almost none evaluated the disparities between urban and rural residents although it seemed very likely that potential socio-demographic and cultural correlates of geriatric depression among an ethnic minority group like Uyghurs would substantially vary between seniors residing in urban and rural areas. Although it was estimated that the prevalence of depression was about 35% among Uyghur (much higher than Han residents) [14], inadequate sample size and other methodological shortcomings precluded the measurement of the actual burden and identification of the potential socio-behavioral correlates of geriatric depression in these studies. Thus, paucity of information on burden and correlates of geriatric depression among Uyghur elderly called for a comprehensive survey involving a representative population so that identification of high risk groups can be made easier and targeted interventions may be planned accordingly to address issues pertaining to the seniors of this minority population residing in urban and rural areas.

This presented manuscript partially reported the findings of our study, while other results from the same survey are reported elsewhere [16].

## Methods

### Recruitment

The current cross-sectional study was conducted in Xinjiang Uyghur Autonomous Region, between the months of March and December, 2011. In order to recruit a representative sample, multistage stratified random sampling was used. Based on the ethnic, demographic, economic and cultural aspects, Xinjiang was divided into three regions (southern, eastern and northern part). In each of these regions an exhaustive list of all cities/counties was prepared and each city was assigned to a unique identification number. Using random number generation tool of Epicalc software, two cities/counties were selected from respective lists in each region. Thus Hotan, Lop, Hami, Huicheng, Urumqi County and Tianshan were selected as the study cities/counties. In the next step from each of these six study cities/counties one community/village was selected randomly following similar random number generation method in Epicalc.

**Figure 1:**
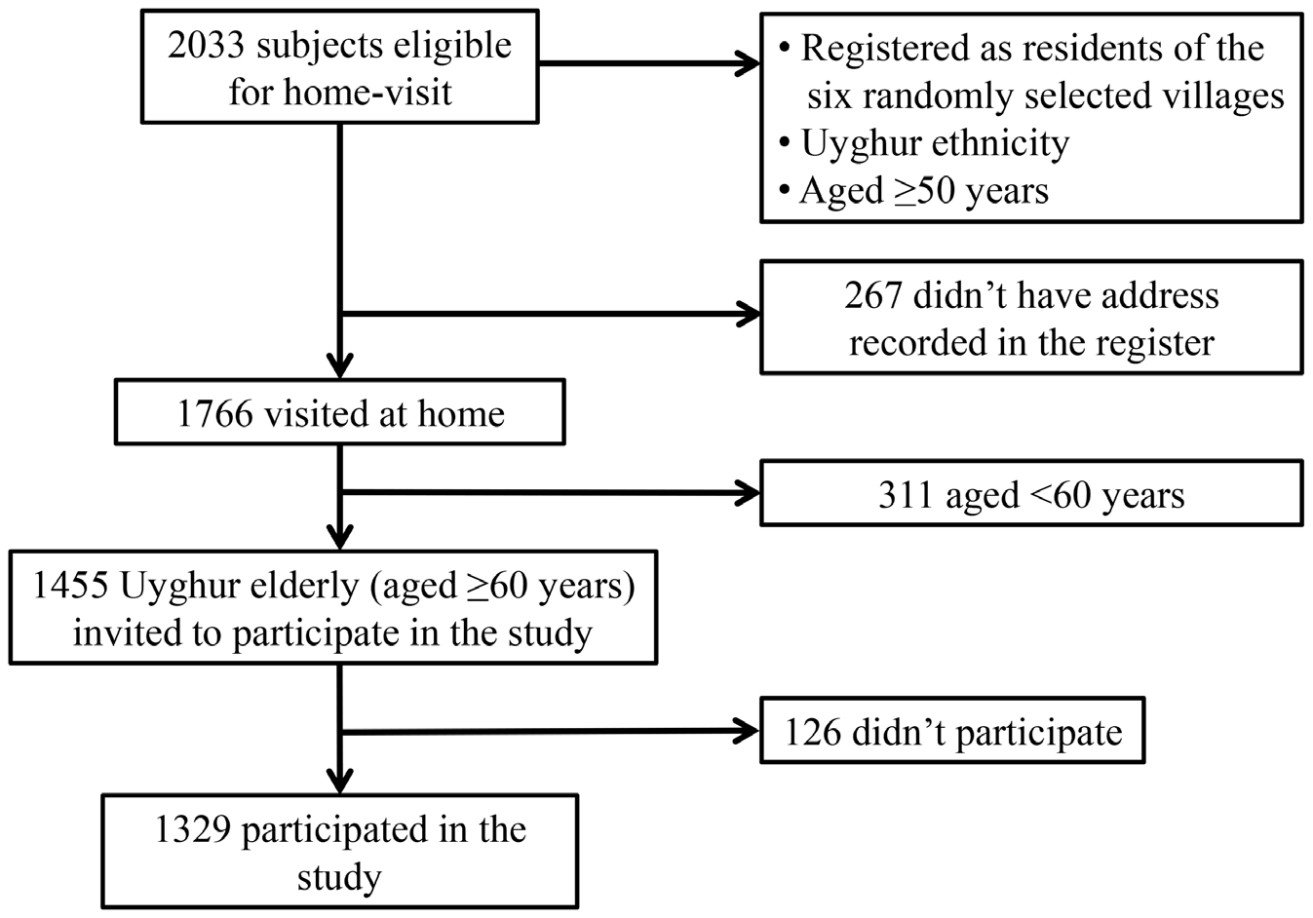
Flow Chart of Home-based Screening of the Elderly Residents of Uyghur Ethnicity in Xinjiang, China.

From the population register it was revealed that a total of 2033 elderly (aged 50 or more) persons of Uyghur ethnicity were living in those six selected villages/communities and among them, 1766 had their detailed demographic information and address recorded in the system. After prior appointments, all these 1766 seniors were visited at home. 1455 of them were invited to participate in the study as they were aged 60 years or more and finally 1329 of them participated in the survey after providing written informed consent. (Figure 1)

None of the eligible subjects, who refused to participate, had to face any negative consequences for not participating in the study.

In our study, the ethnicity of the participants was determined by checking Chinese population registration system; it was further checked by asking the participants.

### Structured Interview

Each participant was interviewed face-to-face at home using an interviewer-administered, structured questionnaire to collect information on socio-demographics, behaviors, chronic diseases and other parameters used for the assessment of geriatric depression.

The demographic information included age (continuous and further categorized into 60–64, 65–69, 70–74, 75 and above), gender (female or male), education level (elementary school or less, junior or senior high school and college or higher), marital status (never married, married, divorced or widowed), residential area (urban or rural), occupation before retirement (officer, worker, farmer or others)and average annual income per person in the family (Yuan, 1 US dollar  = 6.05 Yuan).

In our study, the definition of the residential area was based on the Hukou (residency, which was determined by whether the people are farmers or not) determination method of the Population Registration system of Government of China. In the system, each residence was assigned with either an urban (non-farmers) or a rural Hukou (Famers) [17].

Recent behavior was assessed by collecting information (in a yes/no format) on: smoking, alcohol drinking and religious belief. Satisfaction level of the participants regarding their current income and quality of life (very satisfied, satisfied and dissatisfied) was enquired. History of having one or more chronic diseases like hypertension, cardiovascular diseases, high blood cholesterol, angina, stroke, diabetes, head injury, migraine, meningitis, epilepsy, chronic obstructive pulmonary diseases (COPD), cancer, eye diseases, hearing problem, hyperthyroidism, Parkinson's disease, dementia or kidney disease was collected. We also asked whether the participants had the ability to take care of themselves in daily life and what sorts of common hobbies (watching TV, listening to radio, watching opera or films, reading books or papers, calligraphy, planting flowers, having pets, fishing, playing cards, playing Majiang, playing chess, exercising, walking and group travel) they had.

From a list of events that might have a negative impact on the mind (significant deterioration of health status, serious economic trouble, death of close persons and losing a loved object), subjects were asked to recall how many of these kinds of negative events they had suffered from in the past two years.

To reduce the problem of dimensionality caused by large number of variables representing social contact and support, and to find specific constructs, we created factor scores from those variables. Factor analysis with principal component extraction and varimax rotation method was employed (factor loading ≥0.60) to extract knowledge and access to preventive services. We used orthogonal (varimax) rotation because it could make interpretation simpler and also could maintain independence of the factors [18]. The results extracted from factor analysis represented three underlying factors (based on loading of specific items) for social contact and support. Factor 1 was “living close to good friends' residences and keeping frequent communications with them”, factor 2 was “being able to get help when needed and to be satisfied with that help” and factor 3 was “living close to children, brothers/sisters and other relatives”.

### Geriatric depression assessment

Geriatric Mental State Schedule (GMS) method was used to assess geriatric depression among the subjects. The method of detailed assessment of geriatric depression using this method has been discussed elsewhere [19]. In brief, the data collected from the subjects by using the GMS questionnaire were entered into the Automated Geriatric Examination Computer Assisted Taxon (AGECAT) system, and based on the data, the software automatically provided the diagnostic results. The reported results were divided into five grades, while 0 indicated absence of geriatric depression, 1–2 represented likely (suspected) cases while 3 and 4 referred to confirmed cases. Based on the results of the assessment, participants were classified into those having/not having geriatric depression. The GMS questionnaire also included information regarding self-rated health, physical ailments, social support, life events, hobbies, activities of daily living and self-care ability.

### Data Analysis

Data was double-entered using the software EpiData 3.0 [20] and multiple logic checks were used to ensure the data quality. SAS version 9.1 [21] was used for all statistical analyses. Descriptive analyses were conducted to determine the distribution of the demographic factors, behaviors and to calculate the prevalence proportions [with corresponding 95% confidence intervals (95%CI)] of geriatric depression. In addition, to assess the associations between geriatric depression and their potential correlates, simple logistic regressions were performed using unadjusted (crude) model for bivariate analyses [Odds ratio (OR) and 95%CI]. Based on the literature review age, gender and residence were identified as the most important potential covariates while education, income and marital status were also found to be relevant. Multiple logistic regressions were performed to estimate those associations adjusting (Adjusted odds ratio: AOR and 95%CI) for age (continuous), gender (female/male) and residence (urban/rural) in adjusted multivariate Model 1. In the adjusted multivariate Model 2, education, income and marital status were further adjusted.

### Ethics statement

The study process and content were approved by the Institutional Ethics Committee of the Peoples' Hospital of Xinjiang Autonomous Region, China. Signed informed consent was obtained from each participant prior to the interviews. Each of the participants had the discretion to decline or withdraw from this survey at any point of time. The filled-in questionnaires, written consent documents and computerized data were properly secured.

## Results

### Demographics and disease prevalence

In this comprehensive survey involving Uyghur elderly residents of Xinjiang Uyghur Autonomous Region, a total of 1329 participants were recruited between March and December, 2011. Among these participants, 829 (62.38%) were recruited from urban area and 500 (37.62%) were recruited from rural area. (Table 1)

**Table 1:** Demographics and geriatric depression prevalence among recruited Uyghur elderly of Xinjiang, China (N = 1329).

Variables		Group 1 (n = 829): Urban residents			Group 2 (n = 500): Rural residents			Total (N = 1329)	
		Frequency	Percent	95% CI	Frequency	Percent	95% CI	Frequency	Percent
Age	60–64	457	55.13	51.73,58.52	248	49.6	45.20,54.00	705	53.05
	65–69	178	21.47	18.67,24.27	102	20.4	16.86,23.94	280	21.07
	70–74	119	14.35	11.96,16.75	60	12	9.14,14.86	179	13.47
	75 or older	75	9.05	7.09,11.00	90	18	14.62,21.38	165	12.42
Gender	Male	471	56.82	53.44,60.19	249	49.8	45.40,54.20	720	54.18
	Female	358	43.18	39.80,46.56	251	50.2	45.80,54.60	609	45.82
Marital Status	Married	755	91.07	89.13,93.02	398	79.6	76.06,83.14	1153	86.76
	Never married	33	3.98	2.65,5.31	10	2	0.77,3.23	43	3.24
	Divorced or Widowed	41	4.95	3.47,6.43	92	18.4	14.99,21.81	133	10.01
Education	Elementary school or less	357	43.06	39.69,46.44	367	73.4	69.51,77.29	724	54.48
	Junior or senior high school	211	25.45	22.48,28.42	86	17.2	13.88,20.52	297	22.35
	College or above	261	31.48	28.32,34.65	47	9.4	6.83,11.97	308	23.18
Occupation	Officer	221	26.66	23.64,29.67	25	5	3.08,6.92	246	18.51
	Worker	199	24	21.09,26.92	41	8.2	5.79,10.61	240	18.06
	Farmer	208	25.09	22.13,28.05	412	82.4	79.05,85.75	620	46.65
	Others	201	24.25	21.32,27.17	22	4.4	2.60,6.20	223	16.78
Income (Yuan)	0–3000	562	67.79	64.60,70.98	413	82.6	79.27,85.93	975	73.36
	3001–10000	170	20.51	17.75,23.26	62	12.4	9.50,15.30	232	17.46
	>10000	97	11.7	9.51,13.89	25	5	3.08,6.92	122	9.18
Geriatric depression	No	806	97.23	96.10,98.34	382	76.4	72.66,80.13	1188	89.39
	Yes	23	2.77	1.65,3.89	118	23.6	19.86,27.33	141	10.61

About half of the participants (53.05%) were aged between 60 to 64 years while 12.4% were aged 75 years or more. Among the subjects, 54.2% were male and majority were currently married (86.76%). In our study, approximately 54.5% participants only attended elementary school or less. Before retirement, about 46.6%, 18.1% and 18.5% of participants worked as farmer, worker and officer, respectively. More than three quarters of the participants (73.36%) had an average annual family income of less than 3000 Yuan per person.

Among these participants, 141 (10.61%) met the diagnostic criteria for geriatric depression, while the prevalence proportions among urban and rural area residents were 2.77% (95%CI: 1.65–3.89) and 23.60% (95%CI: 19.86–27.33). Geriatric depression prevalence proportions among male and female were 5.91% and 14.58%, respectively. (Table 1)

### Behaviors and life events

Among the participants, about 6.62% were current smokers, 2.48% were current drinkers, 81.04% [70.57% (95%CI: 67.46–73.68) in urban and 98.40% (95% CI: 97.30–99.50) in rural areas] reported that they were the followers of some religious beliefs.

In this study, 61.93% reported to have one or more diagnosed chronic diseases and 28.37% were suffering from three or more such ailments while this proportion was even higher among rural residents (39.20%, 95%CI: 34.91–43.40).

Majority of the participants (81.64%) reported to engage in some kind of recreational activities while 31.98% [among urban residents: 41.50% (95% CI: 38.13–44.86) and among rural subjects: 16.20% (95%CI: 12.96–19.44)] had three or more hobbies. Recruited subjects mostly (96.16%) mentioned that they could take care of themselves in daily life. About two fifth of the participants (39.28%) experienced at least one negative event (among the listed ones) in the past two years. Among the participating seniors, only 20.69% and 18.36% were very satisfied respectively with their current income and quality of life. (Table 2)

**Table 2:** Behaviors, life events and satisfaction levels among recruited Uyghur elderly of Xinjiang, China (N = 1329).

Variable		Group 1 (n = 829): Urban residents			Group 2 (n = 500): Rural residents			Total (N = 1329)	
		Frequency	Percent	95% CI	Frequency	Percent	95% CI	Frequency	Percent
Smoking status	No	765	92.28	90.46,94.10	476	95.2	93.32,97.08	1241	93.38
	Yes	64	7.72	5.90,9.54	24	4.8	2.92,6.68	88	6.62
Alcohol consumption	No	801	96.62	95.39,97.85	495	99	98.12,99.88	1296	97.52
	Yes	28	3.38	2.14,4.61	5	1	0.12,1.88	33	2.48
Religious belief	No	244	29.43	26.32,32.54	8	1.6	0.50,2.70	252	18.96
	Yes	585	70.57	67.46,73.68	492	98.4	97.30,99.50	1077	81.04
Reported number of diseases	0	413	49.82	46.41,53.23	93	18.6	15.18,22.02	506	38.07
	1	137	16.53	13.99,19.06	104	20.8	17.23,24.37	241	18.13
	2	98	11.82	9.62,14.02	107	21.4	17.79,25.01	205	15.43
	3	181	21.83	19.02,24.65	196	39.2	34.91,43.49	377	28.37
Number of hobbies	0	211	25.45	22.48,28.42	33	6.6	4.42,8.78	244	18.36
	1	103	12.42	10.17,14.67	128	25.6	21.76,29.44	231	17.38
	2	171	20.63	17.87,23.39	258	51.6	47.20,56.00	429	32.28
	3 or more	344	41.5	38.13,44.86	81	16.2	12.96,19.44	425	31.98
Have the ability to take care of themselves in daily life	No	13	1.57	0.72,2.42	38	7.6	5.27,9.93	51	3.84
	Yes	816	98.43	97.58,99.28	462	92.4	90.07,94.73	1278	96.16
Total negative events in the past two years	0	545	65.74	62.50,68.98	262	52.4	48.01,56.79	807	60.72
	1	157	18.94	16.26,21.61	113	22.6	18.92,26.28	270	20.32
	2	79	9.53	7.53,11.53	80	16	12.78,19.22	159	11.96
	3 or more	48	5.79	4.20,7.38	45	9	6.48,11.52	93	7
Satisfaction regarding income	Very satisfied	248	29.92	26.79,33.04	27	5.4	3.41,7.39	275	20.69
	Satisfied	479	57.78	54.41,61.15	365	73	69.10,76.90	844	63.51
	Dissatisfied	102	12.3	10.06,14.54	108	21.6	17.98,25.22	210	15.8
Satisfaction regarding quality of life	Very satisfied	223	26.9	23.88,29.92	21	4.2	2.44,5.96	244	18.36
	Satisfied	456	55.01	51.61,58.40	388	77.6	73.93,81.27	844	63.51
	Dissatisfied	150	18.09	15.47,20.72	91	18.2	14.81,21.59	241	18.13

### Urban and rural disparity

The results of our study demonstrated that rural residents had significantly higher prevalence of geriatric depression (OR = 10.82, 95% CI: 6.8–17.20) compared to their urban counterparts. Similar results were also observed in the age and gender stratified analyses. After adjustment for age, the odds of having geriatric depression among rural residents were about 11 times (AOR = 10.98, 95%CI: 6.90–17.50) that of the urban residents. After further adjustment for gender, the odds were even higher (AOR = 12.66, 95% CI: 7.89–20.33). (Table 3)

**Table 3:** Disparity of geriatric depression between urban and rural Uyghur elderly in Xinjiang, China (N = 1329).

Variable		Urban		Rural		Odds Ratio	95% CI
		Number	Prevalence	Number	Prevalence		
Overall		23/829	2.77	118/500	23.6	10.82	6.81,17.20
Age	*60–64*	11/457	2.41	62/248	25	13.52	6.96, 26.25
	*65–69*	6/178	3.37	27/102	26.47	10.32	4.09,26.03
	*70–74*	4/119	3.36	12/60	20	7.19	2.21,23.41
	*75 and above*	2/75	2.67	17/90	18.89	8.5	1.90,38.12
	*Age adjusted (continuous)*					10.98	6.90,17.50
Gender	*Male*	16/471	3.4	89/249	35.74	15.82	9.02,27.75
	*Female*	7/358	1.96	29/251	11.55	6.55	2.82,15.21
	*Gender adjusted*					12.63	7.88, 20.24
Age and gender adjusted						12.66	7.89,20.33

### Correlates of geriatric depression

Unadjusted model (bivariate analyses) indicated that in the elderly Uyghur population of Xinjiang, the likelihood of having geriatric depression was significantly higher (OR = 10.83, 95%CI 6.81–17.20) among rural (than urban) residents and those having some kind of (compared to those having none) religious belief (OR = 12.20, 95%CI 3.85, 38.61). Having less satisfaction regarding quality of life appeared to be associated with increased odds of developing geriatric depression [with reference to those very satisfied, OR_satisfied_ = 2.16 (95%CI 1.21–3.85) and OR_dissatisfied_ = 2.25 (95%CI 1.16–4.37)]. Compared to those who were not satisfied with their current income, subjects who were satisfied had lower likelihood of having geriatric depression (OR = 0.48, 95%CI: 0.32–0.73).

Number of diagnosed chronic diseases (OR = 2.06, 95% CI 1.75–2.44) and experiencing three or more negative events (compared to no such) in the past two years were positively associated with higher odds of suffering from geriatric depression (OR = 2.56, 95% 1.46–4.44). Lack of ability of self-care was also associated with higher likelihood (OR = 4.22, 95% CI 2.27, 7.84) of having geriatric depression.

The results also pointed out that the Factor 3 (“living close to children, brothers/sisters and other relatives”) was negatively associated (OR = 0.77, 95% CI 0.62–0.96) with geriatric depression. We couldn't find any significant association of smoking, alcohol drinking and two other factors elicited from factor analyses with geriatric depression. (Table 3)

After adjusting for gender, age and residence (adjusted Model 1), alcohol drinking (AOR = 4.28, 95%CI: 1.20–15.32), number of diseases (AOR = 1.70, 95%CI: 1.42–2.04), being unable to take self-care in daily life (AOR = 2.20, 95%CI: 1.09–4.48) and having religious belief (AOR = 3.92, 95%CI: 1.18–13.03) were all positively associated with higher odds of having geriatric depression compared to the corresponding reference groups. Additionally, compared to those who were very dissatisfied, satisfaction regarding quality of life was also negatively associated with geriatric depression (AOR = 0.53, 95%CI: 0.37–0.84). After the adjustment, Factor 1 and Factor 2 were negatively associated with the risk of suffering from geriatric depression, with AORs of 0.63 (95%CI: 0.48–0.82) and 0.77 (95%CI: 0.60–0.99), respectively.

The results were almost unchanged after further adjustment (Model 2) for education, income and marital status. (Table 4)

**Table 4:** Associations of demographic factors, behaviors and other covariates with geriatric depression among Uyghur elderly in Xinjiang, China (N = 1329).

Variable		Crude model		Model 1*		Model 2^#^	
		OR	95% CI	OR	95% CI	OR	95% CI
Satisfaction regarding income	Very satisfied	0.31	0.17,0.56	1.03	0.52,2.07	0.94	0.47,1.89
	Satisfied	0.48	0.32,0.73	0.53	0.37,0.84	0.50	0.32,0.80
	Dissatisfied	Ref		Ref		Ref	
Satisfaction regarding quality of life	Very satisfied	Ref		Ref		Ref	
	Satisfied	2.16	1.21,3.85	0.62	0.32,1.22	0.58	0.29,1.14
	Dissatisfied	2.25	1.16,4.37	0.75	0.35,1.60	0.78	0.36, 1.66
Alcohol drinking	No	Ref		Ref		Ref	
	Yes	1.17	0.40,3.37	4.28	1.20,15.32	4.91	1.41,17.12
Smoking	No	Ref		Ref		Ref	
	Yes	0.71	0.32,1.58	1.62	0.67,3.88	1.71	0.71,4.11
Religious belief	No	Ref		Ref		Ref	
	Yes	12.2	3.85,38.61	3.92	1.18,13.03	3.81	1.14,12.70
Number of diseases		2.06	1.75,2.44	1.70	1.42,2.04	1.70	1.41,2.04
Number of hobbies	0	Ref		Ref		Ref	
	1	1.90	1.05,3.47	0.59	0.30,1.18	0.64	0.32,1.28
	2	1.85	1.08,3.19	0.60	0.32, 1.13	0.61	0.32,1.16
	3 or more	0.96	0.53,1.74	0.78	0.40,1.51	0.77	0.39,1.51
Have the ability to take care of themselves in daily life	Yes	Ref		Ref		Ref	
	No	4.22	2.27,7.84	2.20	1.09,4.48	2.16	1.05,4.43
Total negative events in the past two years	0	Ref		Ref		Ref	
	1	1.19	0.76,1.88	0.93	0.57,1.52	0.94	0.57,1.54
	2	1.34	0.79,2.30	0.95	0.53,1.69	1.00	0.56,1.80
	3	2.54	1.46,4.44	1.72	0.92, 3.22	1.89	1.01,3.56
Factor1 (“living close to good friends' residences and keeping frequent communications with them”)		1.21	0.98,1.50	0.63	0.48,0.82	0.66	0.50,0.86
Factor2 (“being able to get help when needed and to be satisfied with that help”)		1.01	0.81,1.25	0.77	0.60, 0.99	0.78	0.60,0.99
Factor3 (“living close to children, brothers/sisters and other relatives”)		0.77	0.62,0.96	1.06	0.83,1.37	1.10	0.85,1.42

Note: * Model 1 adjusted for age (continuous), gender and residence; ^#^ Model 2 adjusted for age (continuous), gender, residence, education, income and marital status.

## Discussion

In this comprehensive survey involving a representative sample of Uyghur elderly of Xinjiang, China, the prevalence of geriatric depression was found to be 10.61%.

The observed proportion for geriatric depression corroborated with the findings from one previous study conducted among seniors in Beijing [22], however the observed prevalence of the current study was higher than the corresponding findings from a study conducted in Hefei, Anhui [23]. One study conducted among community residents at Karamay District of Xinjiang Uygar Autonomous Region reported a depression prevalence of 43.9% [24], which is much higher than our study. Our study differed in study population (this study only focused on residents in urban area), depression measure method and representative, and these could be the main reasons for the disparity.

In the current study, majority of the patients didn't know that they were suffering from depression. The lack of awareness might have a huge impact on the quality of life of the patients, as these patients might continue to suffer without any medical attention and only at the advanced/incurable stage, medical care would have been sought [25].

In our study, we found that the participants recruited from rural areas had significantly higher prevalence of geriatric depression compared to their urban counterparts. Moreover, corroborating with prior observations, both unadjusted and adjusted model indicated that living in rural area was a potential risk factor for developing geriatric depression [26]. Aged persons living in rural areas were likely to have poorer access to health care services. This might have culminated into increased suffering from chronic diseases resulting in increased likelihood of developing depression [9]. Also the participants living in rural areas might have experienced relatively more difficulties in their daily life compared to the urban residents leading to lower quality of life and less chance of engaging in recreational activities. This in turn probably predisposed in developing depression among the rural elderly. Targeted intervention to improve the healthcare access/utilization and quality of life among Uyghur elderly might be the need of the hour in rural Xinjiang to control geriatric depression.

Alike prior evidences, our study also indicated that with the increase in number of chronic diseases, the risk of developing geriatric depression also increased [27], [28]. This observation might be explained by the possibility that pathogenesis of chronic diseases could have potentially compromised the integrity of the fronto-striatal pathways in the central nervous system and in turn increased vulnerability to depression [9]. Early screening and proper treatment of chronic diseases among elderly could be a potential intervention strategy for the prevention of geriatric depression later on.

Being consistent with previous observations [29], [30], results of the current study emphasized that less satisfaction regarding quality of life and income were highly correlated with depression in old age. Psychosocial handicaps due to these factors might have increased the susceptibility of elderly persons to depression and worsening of its symptoms if acquired early [9]. Being poorly educated, most of our study subjects had higher likelihood of having low awareness and poor quality of life. However, as majority of the participants were married, most of these Uyghur elderly had the option of having support from their spouses to somewhat improve the quality of their life, unless their partners were also sick/depressed.

Besides these, inability to take self-care in daily life was positively correlated with depression. As a potential risk factor for depression [31], this disability could increase the requirement for physical and social support among elderly. While lack of the required support could have increased their susceptibility to depression, perception of this inability (to take own care) itself probably also resulted in hopelessness, anxiety and other psychological disturbances in this population.

Intuitively, participants who experienced three or more negative events in the past two years had higher likelihood of developing depression. Negative events in daily life probably triggered the process of development of depression among them by inducing negative emotion, stress, loneliness and in turn hopelessness.

Religious belief was found to be highly correlated with depression which contradicted some of the previous observations in different religious groups and settings [32], [33]. Persons of Uyghur minority group of Xinjiang, mostly practice Islam which is a minority religious group in China. While factors like government regulation on religious practices and restrictions on religious worships might be potential contributors, reverse causation could well be an explanation if some of the aforementioned correlates and resultant poor self-esteem along with depression had influenced them to become more religious. Further evaluation of this finding with prospective study design might be recommended.

After adjustment for potential confounders, our study indicated that factors “living close to good friends' residences and keeping frequent communications with them” and “being able to get help when needed, and satisfied with the help” were all negatively associated with geriatric depression. These results indicated that better social support from family and friends might potentially reduce the risk of geriatric depression among elderly in this minority group.

Among the participating Uyghur elderly, persons having one or two hobbies were more likely to have depression compared to those who had no hobbies. Reverse causation could be the explanation here also, if loneliness, hopelessness and consequent depression had prompted them to opt for new hobbies.

The strengths of this study include the using of GMS to evaluate the prevalence of geriatric depression, the larger sample size and the potential representativeness of the sample.

There were quite a few potential limitations in our study. Because of the cross-sectional design, temporal ambiguity prevented us from drawing causal inferences based on our results and we recommend that any such interpretation should be made with caution. Due to the same reason, reverse causation was a possible explanation behind some of the observations. Vulnerability of the self-reported information to social desirability bias, might have led to some misclassification in our study. During sampling, 267 Uyghur (aged 50 years or more) residents of the study communities/villages could not be screened for the eligibility as their addresses and other socio-demographic information were not recorded in the population register, while 126 eligible Uyghurs (aged 60 or more) refused to participate. These factors might have introduced the potential for selection bias due to non-response in our study though we expect it to be low because 91.3% of the invited eligible participants finished the survey. As it was not possible for us to have an exhaustive questionnaire, the information collected during the interview only included selected behaviors and covariates leading to the possibilities of residual confounding.

In addition, our study did not collect information on basic activities of daily living and cognitive function, which may further limited our ability to adjust for these possible important confounding factors.

## Conclusion

Despite these potential limitations, by virtue of the representative sample, it can be concluded that the prevalence of geriatric depression in Xinjiang was considerably high among Uyghur elderly. Targeted interventions seemed to be required urgently for controlling the disease in this region, specifically targeting those who are living in rural areas, experiencing more negative events in the past two years, having chronic diseases and not satisfied with their income and quality of life.

## Supporting Information

Dataset S1 **Dataset of Home-based Screening of the Elderly Residents of Uyghur Ethnicity in Xinjiang, China.**
(XLS)
